# OrgTRx: A Platform Developed in Queensland for the Extraction and Visualisation of Antimicrobial Susceptibility Data for the Surveillance of Resistance in Microorganisms

**DOI:** 10.3390/antibiotics15010063

**Published:** 2026-01-06

**Authors:** Sonali Coulter, Holly Hamilton, Philadelphia Holmes, Louise Davis, Claire Heney, David Siebert

**Affiliations:** Pathology Queensland, Royal Brisbane and Women’s Hospital, Herston, QLD 4006, Australia; holly.hamilton@health.qld.gov.au (H.H.); philadelphia.holmes@health.qld.gov.au (P.H.); louisedavisro@gmail.com (L.D.); cgebers@gmail.com (C.H.); david.siebert@health.qld.gov.au (D.S.)

**Keywords:** antimicrobial resistance, AMR, surveillance, antibiograms, dashboards, data science

## Abstract

The OrgTRx platform is a system designed in Queensland, Australia, for the capture of Antimicrobial Resistance (AMR) surveillance data. The data is captured directly from Microbiology Laboratory Information Systems. The most common use of this data is to create facility-specific antibiograms for hospitals and other healthcare facilities. We report on the methods adopted to extract susceptibility results from participating pathology services for AMR surveillance across Australia. OrgTRx receives standardised extracts of antimicrobial susceptibility data from laboratory information systems. This data is validated, verified and incorporated into a database each month. For visualisation by clinical users, the data is displayed in a data cube. The data that is received in this standardised format can be used to review trends in resistance by organism and geographical location of patients presenting with a wide range of infections across Australia. This information can be used to identify areas that require additional resources to combat AMR. The OrgTRx data cube provides clinicians with the tools to create facility-specific antibiograms as well as monitor trends in resistance in pathogens of interest. Increased laboratory capacity and capability, along with adequate funding of surveillance systems, will provide high-quality information to inform the implementation of strategies to prevent the spread of AMR.

## 1. Introduction

Antimicrobial Resistance (AMR) is one of the top 10 global public health concerns, and surveillance of AMR is a key component of the World Health Organization (WHO) global action plan. The action plan on AMR includes intervention from the human, animal and environmental sectors, adopting a one-health approach [1]. A major global study estimated the deaths associated with bacterial AMR in 2019 to be 4.95 million [2].

Australia’s current National AMR Strategy—2020 and beyond—sets out integrated surveillance and response to antimicrobial resistance and usage as its fifth national priority [3]. There are many well-established global surveillance programs to identify critical resistances that are funded by government bodies or pharmaceutical industries. The WHO Global Antimicrobial Resistance Surveillance System (GLASS) is funded by the WHO, and reports on resistance rates aggregated by country. The most recent publication reports on AMR in 127 countries [4]. Surveillance is performed on commonly encountered bacterial pathogens from the bloodstream, gastrointestinal, genital and urinary tract sources. WHONET—developed by the WHO in 1989—is a freely available electronic system that is designed to capture AMR data in human health. The system is used by 130 countries to strengthen their surveillance systems [5].

Another well-established global surveillance program is the Study for Monitoring Antimicrobial Resistance Trends (SMART) surveillance program, which focuses on monitoring Gram-negative pathogens in vitro to identify trends in resistance. This international program began in 2002 and has now captured 20 years of data from 64 countries [6]. SENTRY Antimicrobial Surveillance Program is one of the longest-running global programs established in 1997. SENTRY takes a prevalence-based approach and recruits medical centres and hospitals worldwide to focus on clinical isolates from various body sites, including the bloodstream, skin and soft tissues, urinary tract, and respiratory and intra-abdominal sites, and invasive fungal infections [7]. Other programs include the Central Asian and European Surveillance of Antimicrobial Resistance (CAESAR), the European Antimicrobial Resistance Surveillance Network (EARS-Net), the Latin American Network for Antimicrobial Resistance Surveillance (ReLAVRA) and the UK’s Fleming Fund [8].

Surveillance data can be used in the tracking of significant and life-threatening antimicrobial resistance to commonly prescribed antimicrobials, as well as in identifying areas of changing resistance patterns. This data has many purposes, most significantly the ability to apply acquired information at local, national and global levels to inform treatment guidelines. Surveillance also informs infection control and public health initiatives to target areas of concern.

A recent review found that there are many inconsistencies in how surveillance data is collected, and a lack of information on the methods used to collect this data [9]. The heterogeneity of surveillance data has been noted before. In a review of global surveillance systems, Do et al. [10] found that data infrastructure, quality assurance, non-standardised methods, incomplete data collection, and the engagement of governmental bodies present a prominent issue across the board. Sustainability in terms of the economic elements that are necessary for the function of surveillance was also noted as difficult to secure.

We report the mechanism by which surveillance data are collated in the OrgTRx system, which is a platform designed and developed in Queensland, Australia. This system is used by the Antimicrobial Use and Resistance in Australia (AURA) surveillance program to report on Australian Passive AMR Surveillance (APAS). The data collected are mainly used in the preparation of antibiograms at a facility level and to review trends in resistance of key organism–antimicrobial combinations.

In Australia, participation in national surveillance activities is voluntary. As such, some pathology services do not contribute to APAS, which results in deficiencies in the coverage of surveillance data across Australia. While it is unknown what the exact proportion of laboratories or hospitals contributing to APAS currently is, OrgTRx is used to submit data to GLASS by the AURA program. We estimate that, currently, 525 healthcare facilities and 120 laboratories contribute to GLASS in Australia.

In Australia, as reported in Canada, a centrally managed surveillance system with appropriate governance structures and leadership will facilitate a more sustainable and timely surveillance effort [11]. The UK government ensures that surveillance systems need regular evaluation to ensure that they are operational, efficient and cost-effective [12]. The establishment of the new Australian Centre for Disease Control (ACDC), which is being launched in January 2026, may provide an opportunity to evaluate the existing surveillance system.

OrgTRx uses a data cube (Panorama Necto software version 22.3) to visualise the data and was developed by a multidisciplinary team in Queensland Health (QH) in 2006. The system has continued to evolve over time, and in 2015, it was commissioned by APAS as the platform for the national surveillance program. The national pilot study, involving a single external contributor, commenced in the same year. Since then, 12 additional pathology services have been integrated and contributed longitudinal passive AMR data to OrgTRx.

All participating health services have access to the information they contribute for the preparation of regular local reports and antibiograms to support the antimicrobial stewardship and infection prevention and control teams in the jurisdictions that they serve. In Australia, the Australian Commission on Safety and Quality in Health Care (ACSQHC) is responsible for formulating and administering the National Safety and Quality Health Service (NSQHS) Standards, which are the standards that hospitals must meet for accreditation. The production of antibiograms has been mandated as part of the hospital accreditation program in Australia, where all hospitals are required to have current antibiograms available to their clinical teams for their facility.

Timely access to accurate data is essential to improve decision making in healthcare. The data in OrgTRx is protected from unauthorised use and disclosure by the application of security and access management.

## 2. Results and Reporting Formats

### 2.1. Antibiograms

The data in OrgTRx is mostly used by clinicians to create facility-specific antibiograms. The CLSI M39 guidelines [13] are used as a reference when designing the generic antibiogram workboards in OrgTRx [14]. The updated guidance documents provide instructions on the development of antibiograms, acknowledging that there is variation in data sources, laboratory testing and reporting methodologies.

Panorama Necto software enables the user to select data to include or exclude specific parameters, so that only the information that is relevant is displayed in the workboard. For example, using the age group slicer, the user can create paediatric antibiograms by selecting only the appropriate age range. The antibiograms are set up to include organisms based on frequency of isolation, where a cut off number ≥30 is applied. This cut off removes organisms isolated in low numbers so that the percent susceptible displayed is more robust [15]. There is also a first isolate rule dimension in the data cube that identifies consecutive collections from the same patient through a unique patient identifier. The application of this rule ensures that for the creation of antibiograms, duplicated results caused by multiple presentations from the same patient are removed, therefore eliminating the potential for skewing the data.

Generic workboards have been created and formatted using the colour coding recommended by the CLSI M39 guidelines, where isolates <60% susceptible are colour-coded red, 60–80% yellow and >80% green. Intrinsic resistances displayed by certain organisms are also excluded according to the EUCAST expected resistant phenotype rules and supplemented by CLSI M100 Appendix B recommendations [16]. This enables clinical microbiologists to create their facility-specific antibiograms that are required for hospital accreditation and to inform prescribers of the most suitable empirical therapy options.

**Figure 1:**
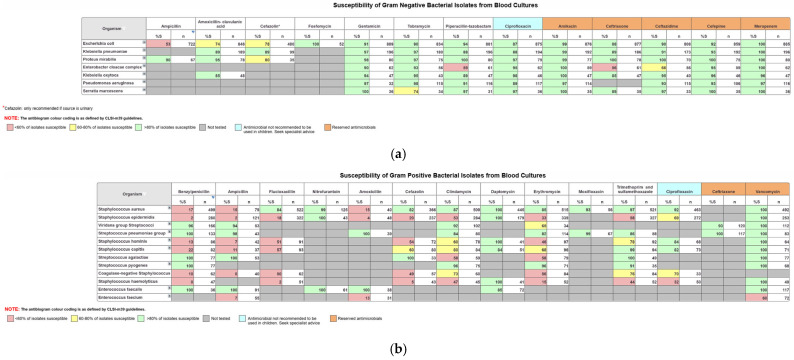
(**a**) Formatted antibiogram for Gram-negative organisms from blood cultures. (**b**) Formatted antibiogram for Gram-positive organisms from blood cultures.

Figure 1a below displays an example of the formatted antibiograms for blood isolates for Gram-negative organisms with relevant antimicrobials, and Figure 1b displays blood isolates for Gram-positive organisms with the relevant antimicrobials. Antibiograms can be created for any selected sample type.

### 2.2. Dashboards

Dashboards are created to inform clinical teams utilising this data for antimicrobial stewardship and research purposes. The QH data science team is interactive and collaborative with clinicians, who, in turn, educate prescribers in reviewing specific resistance trends relevant to their geographic regions. An example of a useful view is one created at the request of a clinician regarding displaying the most frequently isolated pathogens in blood cultures, stratified by location, gender and age group. The general trend in Queensland that was observed in the 20–39-year age group was that the most isolated organism in females was *Escherichia coli*, and in males, it was *Staphylococcus aureus*. However, in the older age group, >65 years, *Escherichia coli* was the most isolated organism for both genders. This information is useful when selecting empirical therapy in an emergency department when a patient presents with a pyrexia of unknown origin.

Clinical users are encouraged to engage and discuss their requirements from a clinical perspective. This interactive process leads to the development of views for a diverse range of clinical purposes. The data in OrgTRx are used to assist in answering specific questions from clinicians routinely treating patients and to assist in decision making for empirical therapy. This interaction also allows for a process to ensure any updates to laboratory practice are noted and considered for national data interpretation.

### 2.3. Geospatial Mapping Capabilities

The patient postcode is included in the data elements extracted from pathology services. The geospatial dashboard displays data by converting patient postcodes into statistical areas (SAs) to provide a location-based analysis of antimicrobial resistance trends. SA mapping is a nested hierarchy of geographies, with each level directly aggregating to the next level, developed by the Australian Bureau of Statistics (ABS). The smallest component is a Mesh Block that generally contains 30–60 dwellings. Being able to create antibiograms for regions based on geography provides additional information on resistance trends in communities. APAS data is now available via a publicly accessible map for specific organism–antimicrobial combinations, displayed by SA3 and SA4 geographic regions. For example, Flucloxacillin resistance in *Staphylococcus aureus* and Ceftriaxone or Cefotaxime resistance in *Escherichia coli* are some of the views available. The number of isolates that comprises the % resistant, and the region is displayed when selecting an area to be observed on the map of Australia.

## 3. Discussion

The management of a complex AMR surveillance database requires clinical knowledge of current laboratory procedures used in a microbiology laboratory to correctly interpret trends observed in the data. Anomalies in the data may be due to the use of different interpretive criteria or reporting processes and, if not accounted for, can lead to misinterpretation of antimicrobial resistance.

This is one of the reasons that it is difficult to compare trends across the globe. The quality of results may vary due to the method of testing performed and the quality control measures applied to the testing. In resource-limited settings, access to state-of-the-art technology for the identification of organisms and susceptibility testing may not be affordable. Additionally, access to trained scientific staff with the level of knowledge to perform the testing could also be scarce. In these environments, the burden of AMR could be significantly underestimated.

In a country like Australia, residents living in scattered, small inland population centres do not have immediate access to the more sophisticated health care services available in large coastal cities. There is often difficulty in the transportation of specimens to the testing laboratory, and this process may take several days to return any results to the treating clinician. While that individual result may not benefit that individual patient, this information is crucial in terms of longitudinal surveillance of AMR. The message that needs to be delivered to clinicians in remote and regional areas is to continue to collect specimens, even if the turnaround time is not always useful for individual patient treatment. Eventually, the database can provide information to assist in identifying pockets of resistance in remote populations across the country. The geospatial mapping tool can be useful in creating antibiograms based on the patients’ postcodes. These maps can provide information on resistance trends in important pathogens in remote areas. The value of this geospatial tool is that it is publicly available for all clinicians to access nationally.

While resource limitations continue to be an issue in remote and regional areas, governments need to ensure that funding is secured for capacity building in laboratory infrastructure and improvements in courier services from remote areas to testing laboratories. Until these needs are prioritised, the barriers to improved healthcare in these areas will continue to exist.

From a clinical perspective, the information in the OrgTRx database is rich and spans many years. The data in Queensland spans over 19 years and has been used for numerous research projects over time. While it is difficult to compare global AMR data due to several variables, as discussed earlier, it is still important to continue collecting available information where possible. There have been requests from clinicians to create the option to capture Minimum Inhibitory Concentration (MIC) values in addition to S, I and R results in OrgTRx. However, since there are several different LISs contributing to the data and not all of them have the capability of reporting MIC values, this remains a difficult task. The data cube and the XML format would also need to be modified to include additional members in the results dimension. While this may be possible in the future, this adaptation has not yet been included in the current scope of the system.

There are several challenges in maintaining a surveillance system from a national perspective. The voluntary nature of contributing to passive surveillance programs will invariably lead to gaps in the data. While standardisation of the data elements is important when receiving data from pathology services using different reporting guidelines and testing methodologies, the system needs to be robust enough to accept the varied information provided.

The establishment of a secure VPN connection from the contributing external pathology services to Queensland Health requires the co-ordination and expertise of Information Technology (IT) teams to ensure that essential data security measures are met. It can be challenging at times to identify and secure the relevant IT resources to ensure connectivity is established and maintained.

After the initial integration of pathology services’ data, technology can evolve to where a connected site might change its existing laboratory information system. This change then requires the re-integration of the new laboratory information system and the merging of new data with the historical data for that specific site. This involves remapping and merging of the location codes so that the data appears seamless. Also, all new codes for organisms, antimicrobials and specimen categories need to be remapped in the database.

While this can be challenging, it is also a rewarding process where the integration of each new laboratory information system provides opportunities for improvement and streamlining the process. Incentives for all pathology services to contribute data for passive surveillance of AMR would be beneficial to further foster engagement and expand national data representation for improved analytical capabilities.

## 4. Materials and Methods

### 4.1. Data Extract

Data from contributing pathology services across Australia are collected directly from each Laboratory Information System (LIS) using locally developed stored procedures and extracted as Extensible Markup Language (XML) files. The XML file is developed using a specification document provided to participants by QH with a data validation tool, designed to ensure that all extracted data fields are formatted according to the database specifications. A three-month rolling extract is forwarded to OrgTRx each month, which includes completed and validated data from the previous two months and the new current month of data. This standardised XML extract is uploaded into the OrgTRx database (staging environment).

Structured Query Language (SQL 2022) scripts in the staging database transform data to a common format and then write to the observation tables. The information associated with the unique laboratory number is used to incorporate the patient-level data from the Laboratory Information System (LIS). The information is divided into two parts: the Observation Order, which contains all the patient-level information associated with the specimen, and the Observation Result, which contains the number of organisms isolated and the individual susceptibility results, as tested and reported by the microbiology laboratory. All antimicrobials tested are captured in the XML extract regardless of whether they are released to the clinicians. The essential data elements included in this extract can be seen in Table 1.

The data extracted and submitted from each LIS includes all validated organisms reported with an antimicrobial susceptibility result. The scope of OrgTRx mainly encompasses bacterial isolates but also contains certain fungal pathogens. 

### 4.2. Data Flow

**Figure 2:**
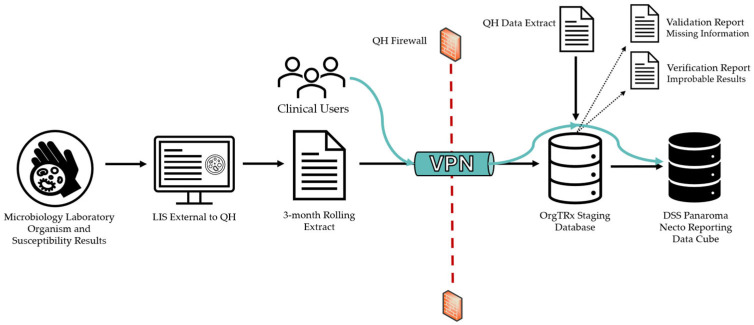
Process flow of data capture and display using OrgTRx.

The files are transferred via a Virtual Private Network (VPN) tunnel using a secure File Transfer Process (sFTP) set up between the contributing site and Queensland Health at the integration of the individual site (Figure 2). Clinical users external to QH can visualise their data using this VPN tunnel to connect to the DSS Panorama Necto reporting data cube.

While all the data received are from National Association of Testing Authorities (NATA) accredited laboratories, the testing methods and susceptibility reporting guidelines can vary between each laboratory, making it difficult to compare susceptibility trends between jurisdictions. In Australia, the European Committee on Antimicrobial Susceptibility Testing (EUCAST) guidelines have been adopted increasingly by the majority of laboratories, and the Clinical & Laboratory Standards Institute (CLSI) guidelines are also currently in use. The susceptibility data captured in OrgTRx are reported in the form of an R (resistant), I (intermediate (CLSI) or susceptible increased exposure (EUCAST)) or S (susceptible). The antibiograms created in OrgTRx use these extracted interpretations to report data as a percent susceptible (%S). When creating antibiograms, laboratories using EUCAST combine S&I to calculate %S, and for the laboratories using CLSI, only S is reported as susceptible.

Additionally, increasing scenarios are occurring where reporting guidelines have introduced different breakpoint interpretations based on drug formulation mechanisms for antimicrobials. Where a contributing pathology service’s laboratory information system has the capability to generate different antimicrobial codes to represent the different breakpoints, OrgTRx receives both codes separately and displays them as two separate entities. For example, Amoxicillin–clavulanic acid, tested and reported for two different breakpoints, has the codes AUGORAL (oral) and AUGIV (IV).

### 4.3. Data Validation

When the data is uploaded into the OrgTRx staging environment each month, a validation job is executed on the new information. The produced validation report checks the integrity, accuracy and structure of the data, and any codes that are not currently defined in the mapping tables are identified. This mapping process is performed in collaboration with the laboratory contact for each individual site and is essential to ensure totality and accuracy of the data prior to incorporation into the data cube.

### 4.4. Data Verification

A verification report is also generated each month. It is a server agent job that processes the new data and identifies any improbable or significant results that meet the parameters outlined in the OrgTRx verification rules. These rules also include organisms with notable, unexpected or pan-resistant phenotypes. Examples would include *Staphylococcus aureus* reported as resistant to Vancomycin, or the isolation of *Candida auris*. These results are sent back to the testing laboratories so that the data can be verified. If an error in laboratory reporting is identified, the erroneous report can be amended, and the corrected data resent in the next month’s extract. This process allows for the quality assurance of the critical reports that are sent out, in addition to ensuring the data in the data cube is of a high quality. All these factors contribute towards the standardisation and the quality assurance of the data that the OrgTRx system receives and presents.

### 4.5. The Panoroma Necto Data Cube

Panorama Necto is an enterprise-grade business intelligence software package, widely used for data visualisation purposes. The data cube has multiple dimensions, and members of these dimensions can be included as slicers of the data. The data can be visualised in tables, charts and infographics. The data cube can be likened to a large pivot table, which can be altered to visualise the specific data parameters as required. Saved public workboards are updated each month as the new data for each of the laboratory services are uploaded into OrgTRx. Access to this data cube is managed by Queensland Health to ensure that each laboratory service’s data is only shared with authorised personnel for that service.

Aggregated de-identified datasets extracted from OrgTRx are used for national reporting by the ACSQHC for the AURA program. This report provides information on overall trends in resistance across Australia of important pathogens to commonly used antimicrobials.

## 5. Conclusions

AMR is an increasing threat to global health and requires urgent human intervention. The OrgTRx platform creates a standardised format review of national AMR data. The system provides clinicians with the tools to predict trends in resistance. The data from APAS is aggregated and reported nationally to inform all clinicians of notable trends in AMR through the AURA program. In addition, the publicly available geospatial map provides access to curated data based on patients’ postcodes. This information can be especially useful in remote areas where there is difficulty in receiving pathology results in a timely manner to directly inform individual patient management.

Passive surveillance systems tap into already existing data, often extensive longitudinal datasets, which is a cost-effective and efficient method to inform empirical therapy and provide better care for patients with serious infections. The investment in local pathology laboratories is crucial for providing reliable, standardised and high-quality information. In addition, appropriate resourcing is required to maintain a robust, sustainable surveillance system that is capable of accepting data from various sources. Information needs to be of a high quality to appropriately inform the implementation of the national AMR strategy.

OrgTRx, as a platform, is designed to ingest validated results used for longitudinal review of passive AMR. For this purpose, the data is only incorporated once a month. While there would be a benefit in introducing the capabilities for real time validated data, there would need to be considerable infrastructure changes for this to be feasible. It is important to ensure that appropriate clinical stakeholders are engaged when reviewing an established surveillance program. Surveillance systems will always require refinement to meet the ever-changing surveillance objectives.

## Figures and Tables

**Table 1:** Data elements.

Observation Order	Observation Result
Patient Code–Unique Identifier	Organism
Date of Birth	Organism Number
Sex	Antimicrobial
Patient Category	Susceptibility
Post Code	Testing Laboratory
Facility	
Ward	
Order Number	
Date Collected	
Primary Site	
Specimen Category	

## Data Availability

The data is unavailable due to data governance constraints for each contributing site.

## Abbreviations

The following abbreviations are used in this manuscript:

AMR	Antimicrobial Resistance
WHO	World Health Organization
GLASS	Global Antimicrobial Resistance Surveillance System
SMART	Study for Monitoring Antimicrobial Resistance Trends
APAS	Australian Passive AMR Surveillance
AURA	Antimicrobial Use and Resistance in Australia
QH	Queensland Health
ACSQHC	Australian Commission on Safety and Quality in Health Care
NSQHS	National Safety and Quality Health Care
LIS	Laboratory Information System
XML	Extensible Markup Language
VPN	Virtual Private Network
sFTP	Secure File Transfer Process
NATA	National Association of Testing Authorities
EUCAST	The European Committee on Antimicrobial Susceptibility Testing
CLSI	Clinical & Laboratory Standards Institute

## References

[1] Tang K.W.K., Millar B.C., Moore J.E. (2023). Antimicrobial Resistance (AMR). Br. J. Biomed. Sci..

[2] Collaborators A.R. (2022). Global burden of bacterial antimicrobial resistance in 2019: A systematic analysis. Lancet.

[3] Australian Government, Department of Health, Department of Agriculture, Water and the Environment (2020). Australia’s National Antimicrobial Resistance Strategy 2020 & Beyond.

[4] WHO (2022). Global Antimicrobial Resistance and Use Surveillance System (GLASS) Report: 2022.

[5] Oberin M., Badger S., Faverjon C., Cameron A., Bannister-Tyrrell M. (2022). Electronic information systems for One Health surveillance of antimicrobial resistance: A systematic scoping review. BMJ Glob. Health.

[6] Cantón R., Gottlieb T., Coombs G.W., Woo P.C.Y., Korman T.M., Garcia-Castillo M., Daley D., Bauer K.A., Wong M., Wolf D.J. (2023). Antimicrobial surveillance: A 20-year history of the SMART approach to addressing global antimicrobial resistance into the future. Int. J. Antimicrob. Agents.

[7] Fuhrmeister A.S., Jones R.N. (2019). The Importance of Antimicrobial Resistance Monitoring Worldwide and the Origins of SENTRY Antimicrobial Surveillance Program. Open Forum Infect. Dis..

[8] Frost I., Kapoor G., Craig J., Liu D., Laxminarayan R. (2021). Status, challenges and gaps in antimicrobial resistance surveillance around the world. J. Glob. Antimicrob. Resist..

[9] Tacconelli E., Sifakis F., Harbarth S., Schrijver R., van Mourik M., Voss A., Sharland M., Rajendran N.B., Rodríguez-Baño J., Bielicki J. (2018). Surveillance for control of antimicrobial resistance. Lancet Infect. Dis..

[10] Do P.C., Assefa Y.A., Batikawai S.M., Reid S.A. (2023). Strengthening antimicrobial resistance surveillance systems: A scoping review. BMC Infect. Dis..

[11] Otto S.J.G., Haworth-Brockman M., Miazga-Rodriguez M., Wierzbowski A., Saxinger L.M. (2022). Integrated surveillance of antimicrobial resistance and antimicrobial use: Evaluation of the status in Canada (2014–2019). Can. Vet. J..

[12] Bennani H., Cornelsen L., Stärk K.D.C., Häsler B. (2021). Characterisation and mapping of the surveillance system for antimicrobial resistance and antimicrobial use in the United Kingdom. Vet. Rec..

[13] (2022). Analysis and Presentation of Cumulative Antimicrobial Susceptibility Test Data.

[14] Simner P.J., Hindler J.A., Bhowmick T., Das S., Johnson J.K., Lubers B.V., Redell M.A., Stelling J., Erdman S.M. (2022). What’s New in Antibiograms? Updating CLSI M39 Guidance with Current Trends. J. Clin. Microbiol..

[15] Tran C., Hargy J., Hess B., Pettengill M.A. (2023). Estimated Impact of Low Isolate Numbers on the Reliability of Cumulative Antibiogram Data. Microbiol. Spectr..

[16] (2026). Performance Standards for Antimicrobial Susceptibility Testing.

