# Correction: Coulter et al. OrgTRx: A Platform Developed in Queensland for the Extraction and Visualisation of Antimicrobial Susceptibility Data for the Surveillance of Resistance in Microorganisms. *Antibiotics* 2026, *15*, 63

**DOI:** 10.3390/antibiotics15060613

**Published:** 2026-06-17

**Authors:** Sonali Coulter, Holly Hamilton, Philadelphia Holmes, Louise Davis, Claire Heney, David Siebert

**Affiliations:** Pathology Queensland, Royal Brisbane and Women’s Hospital, Herston, QLD 4006, Australia; holly.hamilton@health.qld.gov.au (H.H.); philadelphia.holmes@health.qld.gov.au (P.H.); louisedavisro@gmail.com (L.D.); cgebers@gmail.com (C.H.); david.siebert@health.qld.gov.au (D.S.)

## Text Correction

There has been a request from the Australian Commission for Safety and Quality in Health Care to include an amendment note for this paper [1].

The Australian Commission on Safety and Quality in Health Care (ACSQHC; The Commission) is funded by the Australian Centre for Disease Control (from 1 January 2026; previously the Australian Government Department of Health, Disability and Ageing) for the coordination on Australian Passive AMR Surveillance (APAS) as part of the AURA surveillance program. The Commission established APAS in 2015 in collaboration with Queensland Health and it currently operates under a subcontract arrangement with Queensland Health for access, support and maintenance of OrgTRx.

The Commission routinely publishes data analysis reports on aggregate national APAS data on relevant AMR topics for patient safety or emerging issues and developed the publicly accessible APAS Data Explorer dashboard map.

The Commission has also developed the National Safety and Quality Health Service (NSQHS) Standards in collaboration with the Australian Government, states and territories, the private sector, clinical experts, patients and carers. The NSQHS Standards provide a quality assurance mechanism that tests whether relevant systems are in place to ensure that expected standards of safety and quality are met to protect the public from harm and improve the quality of health service provision in Australia. Health services are assessed on meeting the NSQHS Standards by independent accrediting agencies.

The Preventing and Controlling Infections Standard includes actions to prevent and contain AMR and for antimicrobial stewardship programs, for which health services may use antibiograms to inform the development of local empiric antimicrobial prescribing guidelines and formulary management and monitor local resistance trends over time.

The authors state that the scientific conclusions are unaffected. This correction was approved by the Academic Editor. The original publication has also been updated.
